# Cell adhesion-mediated mitochondria transfer contributes to mesenchymal stem cell-induced chemoresistance on T cell acute lymphoblastic leukemia cells

**DOI:** 10.1186/s13045-018-0554-z

**Published:** 2018-01-22

**Authors:** Jiancheng Wang, Xin Liu, Yuan Qiu, Yue Shi, Jianye Cai, Boyan Wang, Xiaoyue Wei, Qiong Ke, Xin Sui, Yi Wang, Yinong Huang, Hongyu Li, Tao Wang, Ren Lin, Qifa Liu, Andy Peng Xiang

**Affiliations:** 1 Program of Stem Cells and Regenerative Medicine, Affiliated Guangzhou Women and Children’s Hospital, Zhongshan School of Medicine, Sun Yat-Sen University, Guangzhou, 510080 China; 2 Center for Stem Cell Biology and Tissue Engineering, Key Laboratory for Stem Cells and Tissue Engineering, Ministry of Education, Sun Yat-Sen University, 74# Zhongshan 2nd Road, Guangzhou, Guangdong China; 3 Biotherapy Center, the Third Affiliated Hospital, Sun Yat-Sen University, Guangzhou, 510080 China; 4 The First Affiliated Hospital of Xi’an Jiaotong University Medical College, Xi’an, Shaanxi 710061 China; 5 Department of Hematology, Nanfang Hospital, Southern Medical University, Guangzhou, 510515 China; 6 Key Laboratory of Protein Modification and Degradation, School of Basic Medical Sciences, Affiliated Cancer Hospital and Institute of Guangzhou Medical University, Guangzhou, 511436 China; 7 Department of Biochemistry, Zhongshan School of Medicine, Sun Yat-Sen University, Guangzhou, 510080 China

**Keywords:** Mesenchymal stem cells, Cell adhesion, Mitochondria transfer, Reactive oxygen species, Chemoresistance

## Abstract

**Background:**

Despite the high cure rate of T cell acute lymphoblastic leukemia (T-ALL), drug resistance to chemotherapy remains a significant clinical problem. Bone marrow mesenchymal stem cells (MSCs) protect leukemic cells from chemotherapy, but the underlying mechanisms are poorly understood. In this study, we aimed to uncover the mechanism of MSC-induced chemoresistance in T-ALL cells, thus providing a promising clinical therapy target.

**Methods:**

Cell viability was determined using the viability assay kit CCK-8. The mitochondrial ROS levels were detected using the fluorescent probe MitoSOX™ Red, and fluorescence intensity was measured by flow cytometry. In vitro, MSCs and Jurkat cells were cocultured. MSCs were labeled with green fluorescent protein (GFP), and Jurkat cells were labeled with the mitochondria-specific dye MitoTracker Red. Bidirectional mitochondrial transfer was detected by flow cytometry and confocal microscopy. The mechanism of mitochondria transfer was analyzed by inhibitor assays. Transcripts related to Jurkat cell/MSC adhesion in the coculture system were assessed by qRT-PCR. After treatment with a neutralizing antibody against a key adhesion molecule, mitochondria transfer from Jurkat cells to MSCs was again detected by flow cytometry and confocal microscopy. Finally, we verified our findings using human primary T-ALL cells cocultured with MSCs.

**Results:**

Chemotherapeutic drugs caused intracellular oxidative stress in Jurkat cells. Jurkat cells transfer mitochondria to MSCs but receive few mitochondria from MSCs, resulting in chemoresistance. This process of mitochondria transfer is mediated by tunneling nanotubes, which are protrusions that extend from the cell membrane. Moreover, we found that most Jurkat cells adhered to MSCs in the coculture system, which was mediated by the adhesion molecule ICAM-1. Treatment with a neutralizing antibody against ICAM-1 led to a decreased number of adhering Jurkat cells, decreased mitochondria transfer, and increased chemotherapy-induced cell death.

**Conclusions:**

We show evidence that mitochondria transfer from Jurkat cells to MSCs, which is mediated by cell adhesion, may be a potential therapeutic target for T-ALL treatment.

**Electronic supplementary material:**

The online version of this article (10.1186/s13045-018-0554-z) contains supplementary material, which is available to authorized users.

## Background

Medical advances have improved the survival of adult patients with acute lymphoblastic leukemia (ALL) over the past few decades. T cell acute lymphoblastic leukemia (T-ALL) is one of the most aggressive hematologic malignancies, accounting for up to 10–15% of pediatric ALL and 25% of adult ALL cases [1], and arises from the malignant transformation of T cell progenitors [2, 3]. Although high-dose multi-agent chemotherapy is clinically effective in most cases, primary drug resistance and relapse are frequently observed [4], preventing T-ALL from being cured [5, 6].

Recent studies have shown that the bone marrow milieu, especially mesenchymal stem cells (MSCs), has pro-survival effects on leukemia cells and protects leukemic cells from chemotherapy [7–11]. On one hand, soluble factor-mediated drug resistance has been proposed to contribute to MSC-induced chemoresistance. Iwamoto and colleagues found that MSCs secreted asparagine that was taken up by ALL cells and thus protected ALL cells from asparaginase treatment [12]. On the other hand, cell adhesion-mediated drug resistance is also an important mechanism of MSC-induced chemoresistance. For example, Mudry et al. reported that MSCs interacted with leukemia cells by increasing the expression of vascular cell adhesion molecule-1 (VCAM-1), protecting leukemia cells from cytarabine and etoposide cytotoxicity [13]. However, the role of MSCs in T-ALL cell drug resistance remains unclear, and thus, intensive studies on the mechanisms by which MSCs protect T-ALL cells are needed to develop T-ALL treatments.

Excessive intracellular reactive oxygen species (ROS) can induce the apoptosis of cells [14–16]. An important mechanism for chemotherapeutic agents is to induce cancer cell apoptosis by enhancing intracellular ROS levels. Such agents include paclitaxel, anthracyclines, ara-C, and methotrexate (MTX), among others [17]. Mitochondria are the most important source of cellular ROS [18–22]. Therefore, upregulating mitochondrial ROS levels is a potential strategy for killing cancer cells [23, 24], including T-ALL cells [25]. Jitschin et al. reported that induction of mitochondrial ROS in chronic lymphocytic leukemia (CLL) cells with PK11195, a drug that can generate mitochondrial superoxide, resulted in cell apoptosis [26]. Our previous research showed that MSCs reduced mitochondrial ROS levels in T-ALL cells through the ERK pathway and thus protected T-ALL cells from chemotherapeutics ara-C or MTX. Accordingly, inhibition of the ERK activation with the ERK inhibitor PD325901 increased mitochondrial ROS levels and the cell death rate of T-ALL cells [27]. These results indicated that MSCs protect T-ALL cells by decreasing mitochondrial ROS levels in T-ALL cells. Interestingly, in the past few years, several studies have reported that mitochondria can move between cells, through tunneling nanotubes (TNTs), microvesicles, or gap junctions, leading to protection against tissue injury or resistance to therapeutic agents [21, 28–37]. However, there are few studies on the mechanisms of mitochondria transfer between MSCs and T-ALL cells. As mitochondrial ROS plays a major role in the intracellular redox balance [38], it is important to determine whether mitochondrial transfer can be used to modify ROS levels.

In this study, we examined bidirectional mitochondria transfer between MSCs and T-ALL cells and found that T-ALL cells exposed to chemotherapeutic drugs transferred many mitochondria to MSCs but received few from MSCs. This process facilitates the proliferation and survival of leukemia cells by reducing ROS levels. Furthermore, by impeding cell adhesion, the mitochondria transfer was disturbed, therefore decreasing the survival rate under chemotherapy. These results suggest that mitochondria transfer may be a candidate target for T-ALL treatment.

## Methods

### Cell culture

Human T-ALL cell line Jurkat was purchased from the Cell Bank of the Chinese Academy of Sciences (Shanghai, China). The culture medium was RPMI 1640 (Hyclone, Logan, UT, USA) supplemented with fetal bovine serum (FBS; Gibco, Grand Island, NY, USA), penicillin, and streptomycin (Sigma, St. Louis, MO, USA).

For collection of human primary T-ALL cells, 10 enrolled T-ALL patients were previously untreated and newly diagnosed at the Department of Haematology, Nanfang Hospital, Southern Medical University (Guangzhou, China), and Department of Pediatrics, Sun Yat-Sen Memorial Hospital, Sun Yat-Sen University (Guangzhou, China). Consent was provided according to the Declaration of Helsinki. Informed consent was obtained following institutional guidelines, and approval was obtained from the institutional review board of Sun Yat-Sen University. All human bone marrow or peripheral blood samples were obtained with written informed consent. Primary CD3+ T-ALL cells were isolated through density gradient centrifugation on standard Ficoll-HyPaque and subjected to fluorescence-activated cell sorting (FACS; BD Bioscience Influx, Franklin Lakes, NJ, USA).

MSCs were collected from bone marrow aspirates of healthy volunteers with informed consent. Isolation and characterization of MSCs were performed as we previously described [39, 40]. Briefly, the bone marrow aspirates were diluted, have undergone the density gradient centrifugation, and were counted and planted before purification [41, 42]. The culture medium was low-glucose DMEM (Hyclone, Logan, UT, USA) supplemented with 10% FBS and 100 IU/ml penicillin and streptomycin. To generate GFP-labeled MSCs, MSCs were transfected with lentivirus containing lentiviral expression vector pLV/puro-EF1a-GFP [43] using the X-treme GENE HP reagent (Roche) according to the manufacturer’s instructions. Three days after transfection, GFP-labeled MSCs were purified by FACS (Influx, Becton Dickinson).

Several culture models were used in this article. (1) Monoculture: Jurkat cells/human primary T-ALL cells (5 × 10^5^ /ml) or MSCs (5 × 10^4^ /ml) were respectively seeded in 24-well plates. (2) Coculture: MSCs (5 × 10^4^ /ml) and Jurkat cells/human primary T-ALL cells (5 × 10^5^ /ml) were suspended in RPMI 1640 and seeded in 24-well plates. (3) Transwell: Jurkat cells/human primary T-ALL cells (5 × 10^5^ /ml) were seeded in the Transwell inserts (Millipore), which were inserted into the 24-well plates with preseeded MSCs (5 × 10^4^ /ml). The relative measurements were performed after coculture for 1 to 3 days.

### Reagents and antibodies

Ara-C and MTX were purchased from Pharmacia Pty Ltd. (NSW, Australia) and Calbiochem (San Diego, CA, USA), respectively. 300nM ara-C or 100 nM MTX were used to cause cytotoxicity in Jurkat cells. 18-α-GA, dynasore, and cytochalasin D were purchased from Sigma-Aldrich and were used in a concentration of 50, 50, and 1 μM, respectively. Neutralizing anti-ICAM-1 antibody (MS305PABX) was purchased from Invitrogen (Carlsbad, CA, USA). Alexa Fluor™ 647 phalloidin was purchased from Thermo Fisher Scientific.

### Cell viability assay

Cell viability was determined using a CCK-8 assay kit (Dojindo Laboratories, Kumamoto, Japan) according to the manufacturer’s instructions. The principle of this assay is that some components of the CCK-8 assay kit will be reduced by mitochondria to produce formazan so as to be detectable. Briefly, 5 × 10^4^ Jurkat cells in 100 μl culture media were plated to a 96-well plate in suspension. Then, the samples were incubated with CCK-8 solution (10 μl) for 4 h at 37 °C; the absorbance in each well was quantified at 450 nm using an automated enzyme-linked immunosorbent assay reader (Tecan, Salzburg, Austria). Cell viability was calculated according to the manufacturer’s instructions.

### Fluorescence staining of mitochondria

MitoTracker Red (Molecular Probes) was used to label mitochondria. Jurkat cells or MSCs were incubated with 200 nM MitoTracker Red in culture media for 10 min at 37 °C. Excess of the dye was washed out with PBS. Then, 4 days later, stained cells were then seeded for monoculture and coculture. We verified the feasibility of mitochondria dye method in Additional file 1: Figure S1.

### Staining of F-actin in MSCs

To visualize TNTs which consist of F-actin, MSCs were immersion-fixed in 4% paraformaldehyde. After a brief permeabilization with 0.1% Triton X-100, cell coverslips were incubated in AlexaFluor 647-conjugated phalloidin for 20 min at room temperature.

### Mitochondrial ROS assessment

The levels of mitochondrial ROS were detected using the fluorescent probes MitoSOX™ Red (Molecular Probes, Life Technologies, Carlsbad, CA, USA), and fluorescent intensity was measured by flow cytometry (FACScan; Becton Dickinson, San Diego, CA, USA).

### Annexin V/PI flow cytometry analysis

Jurkat cells from monoculture or coculture system were treated with ara-C or MTX for 2 days and harvested by centrifugation. Jurkat cells were then stained with annexin V/propidium iodide (PI) assay kit (BIOSCI BIOTECH, Shanghai, China) according to the manufacturer’s instruction. The apoptotic population was immediately evaluated by flow cytometry. The percentages of early apoptotic cells (annexin V^+^/PI^−^) and late apoptotic cells (annexin V^+^/PI^+^) were analyzed and graphed.

### RNA isolation and qRT-PCR analysis

Total mRNA from MSCs was extracted using an RNeasy Mini Kit (Qiagen), and complementary DNA (cDNA) was synthesized using a QuantiTect Reverse Transcription Kit (Qiagen) according to the manufacturers’ protocols. qRT-PCRs were carried out using SYBR Green qPCR SuperMix (Roche, Indianapolis, IN, USA) and a LightCycler 480 Detection System (Roche) as described by the manufacturer. Target mRNA levels were normalized with respect to those of β-actin. The primer sequences used for qRT-PCR are listed in Additional file 1: Table S1.

### Statistical analyses

All experiments were performed at least three separate times. All data are expressed as the mean ± S.E.M. Comparisons among groups were performed using one-way analysis of variance (ANOVA) or Student’s *t* test. Statistical differences were determined by GraphPad Prism 5.0 software (GraphPad Software Inc., CA, USA). A two-sided *P* value < 0.05 was considered to be statistically significant.

For the other experimental procedures, please see Additional file 1.

## Results

### Jurkat cells transfer mitochondria to MSCs when exposed to chemotherapeutic drugs

**Fig. 1:**
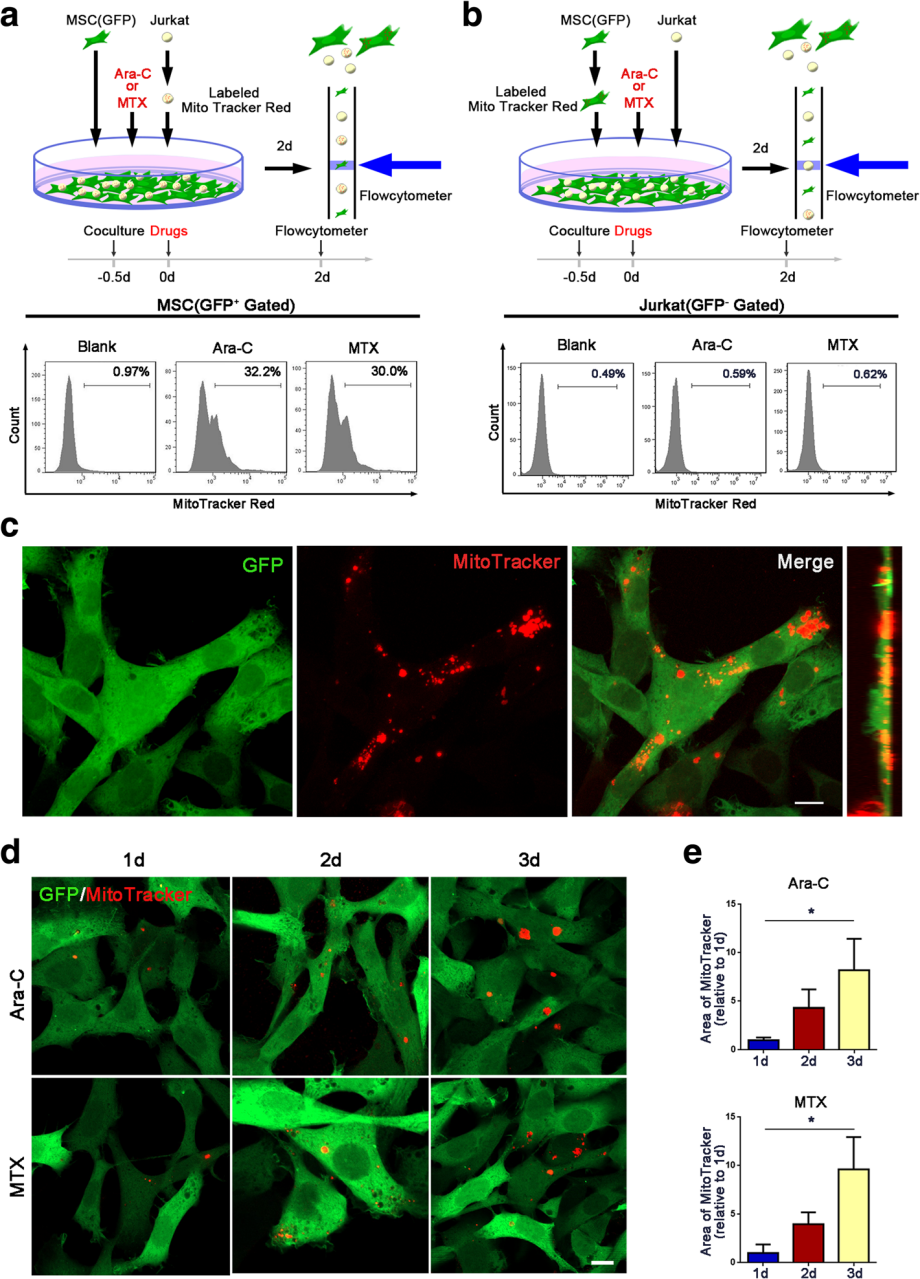
Jurkat cells transfer mitochondria to MSCs when exposed to ara-C or MTX. **a** Flow cytometry analysis of MitoTracker Red uptake by MSCs (GFP+ gated) cocultured with MitoTracker Red-labeled Jurkat cells after 300 nM ara-C or 100 nM MTX was added for 48 h. **b** Flow cytometry analysis of MitoTracker Red uptake by Jurkat cells (GFP− gated) cocultured with MitoTracker Red-labeled GFP+ MSCs after 300 nM ara-C or 100 nM MTX was added for 48 h. **c** Representative confocal microscopy images show that Jurkat cell-derived mitochondria (Red+) were internalized in MSCs(GFP+). Scale bar, 10 μm. **d** Representative confocal images show that MSCs received Jurkat cell-derived mitochondria at different time points (1, 2, and 3 days after coculture). Scale bar, 10 μm. **e** The areas of red foci per field were calculated by ImageJ software. The data are presented as the mean ± S.E.M. of three independent experiments (*P < 0.05; **P < 0.01; *t* test)

We previously found that MSCs could protect T-ALL cells from chemotherapeutic cell death in indirect (Transwell) and direct coculture system. Furthermore, we showed that exposure of T-ALL cells to MSCs decreased mitochondrial ROS levels via the ERK/Drp1 pathway under both culture conditions, However, when exposed to chemotherapeutic drugs, Jurkat cells in direct contact with MSCs exhibited significantly lower mitochondrial ROS levels than cells in the Transwell system [27]. We thus wondered whether there were other mechanisms by which MSCs decrease ROS levels in Jurkat cells in a cytotoxic environment. As mitochondria are the key source of intracellular ROS, alterations in mitochondrial number and function could influence the intracellular ROS levels. We thus explored whether mitochondria transfer occurred between MSCs and Jurkat cells and participated in MSC-induced leukemia cell chemoresistance. First, MSCs were labeled with green fluorescent protein (GFP) by lentiviral transduction to distinguish them from Jurkat cells in the coculture system. These cells were then purified via fluorescence-activated cell sorting (FACS). Prior to coculture experiments, we also labeled MSCs and Jurkat cells with the mitochondria-specific dye MitoTracker Red to observe mitochondria transfer between MSCs and Jurkat cells. Twelve hours later, 300 nM ara-C or 100 nM MTX was added to the coculture system. After 2 days of coculture, we quantified mitochondria transfer by flow cytometry. The results showed that 32.20 ± 5.21% (ara-C-treated group) or 30.00 ± 4.31% (MTX-treated group) of GFP-labeled MSCs were Red+, indicating that approximately 30% of the MSCs received mitochondria from Jurkat cells (Fig. 1a). We also stained GFP-labeled MSCs with MitoTracker Red before coculture with Jurkat cells. However, only 0.59 ± 0.14% (ara-C-treated group) or 0.62 ± 0.15% (MTX-treated group) of the Jurkat cells were Red+ after 2 days of coculture, indicating that few Jurkat cells received mitochondria from MSCs (Fig. 1b). Taken together, these results showed that Jurkat cells could transfer mitochondria to MSCs when treated with chemotherapeutic drugs. We further performed confocal microscopy to directly observe mitochondria transfer. We first labeled Jurkat cells with MitoTracker Red before coculture with GFP-labeled MSCs. After 3 days of coculture, specific fields of view as well as side views of confocal imaging showed that mitochondrial Red fluorescence was internalized in GFP-labeled MSCs (Fig. 1c). In addition, the areas of red foci in GFP-labeled MSCs increased in a time-dependent manner from day 1 to day 3 (Fig. 1d, e), indicating that mitochondria transfer from Jurkat cells to MSCs was dynamic.

### Jurkat cells transfer mitochondria to MSCs via intercellular tunneling nanotubes (TNTs)

**Fig. 2:**
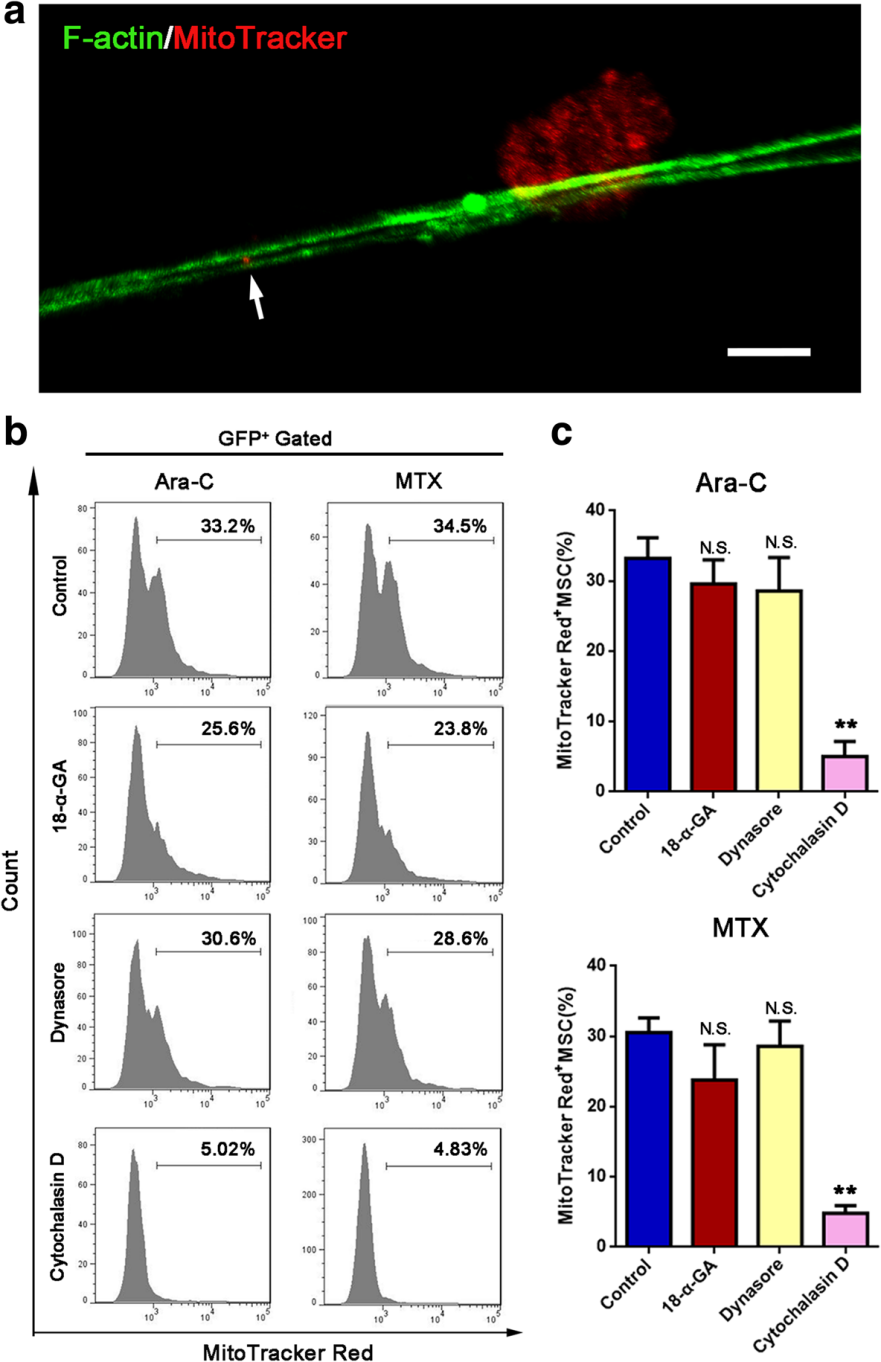
Mitochondria transfer from Jurkat cells to MSCs can be blocked by cytochalasin D. **a** Representative confocal microscopy images show the presence of TNTs containing mitochondria (arrow). Scale bar, 5 μm. **b** 18-α-GA (50 μM), dynasore (50 μM), or cytochalasin D (1 μM) was added to the coculture system with ara-C or MTX for 48 h. Flow cytometry analysis of Jurkat cell-derived mitochondria uptake by MSCs (GFP+ gated). **c** The percentage of Red+ MSCs in each group was analyzed and graphed. The results are expressed as the mean ± S.E.M. of three independent experiments (*P < 0.05; **P < 0.01; *t* test)

Mitochondria transfer between cells has been reported to be mediated by tunneling nanotubes (TNTs), microvesicles and gap junction [36]. In our study, mitochondria transfer through TNTs was confirmed by the presence of TNT containing mitochondria in it (Fig. 2a). Then, 18-α-GA (a blocker of gap junctions), the dynamin inhibitor dynasore (a blocker of microvesicle endocytosis), and the potent actin polymerization inhibitor cytochalasin D (a blocker of TNT formation) were added to the coculture system with ara-C or MTX. Mitochondria transfer was then analyzed with flow cytometry. Prior to coculture, Jurkat cells were stained with MitoTracker Red to label mitochondria. We observed that, compared with DMSO, cytochalasin D significantly decreased the number of Red+ MSCs, whereas 18-α-GA or dynasore treatment had no significant effect on the number of Red+ MSCs (Fig. 2b, c). Therefore, these data show that TNTs are the key mechanism by which mitochondria transfer from Jurkat cells to MSCs.

### Inhibition of mitochondria transfer decreases MSC-induced chemoresistance in Jurkat cells

**Fig. 3:**
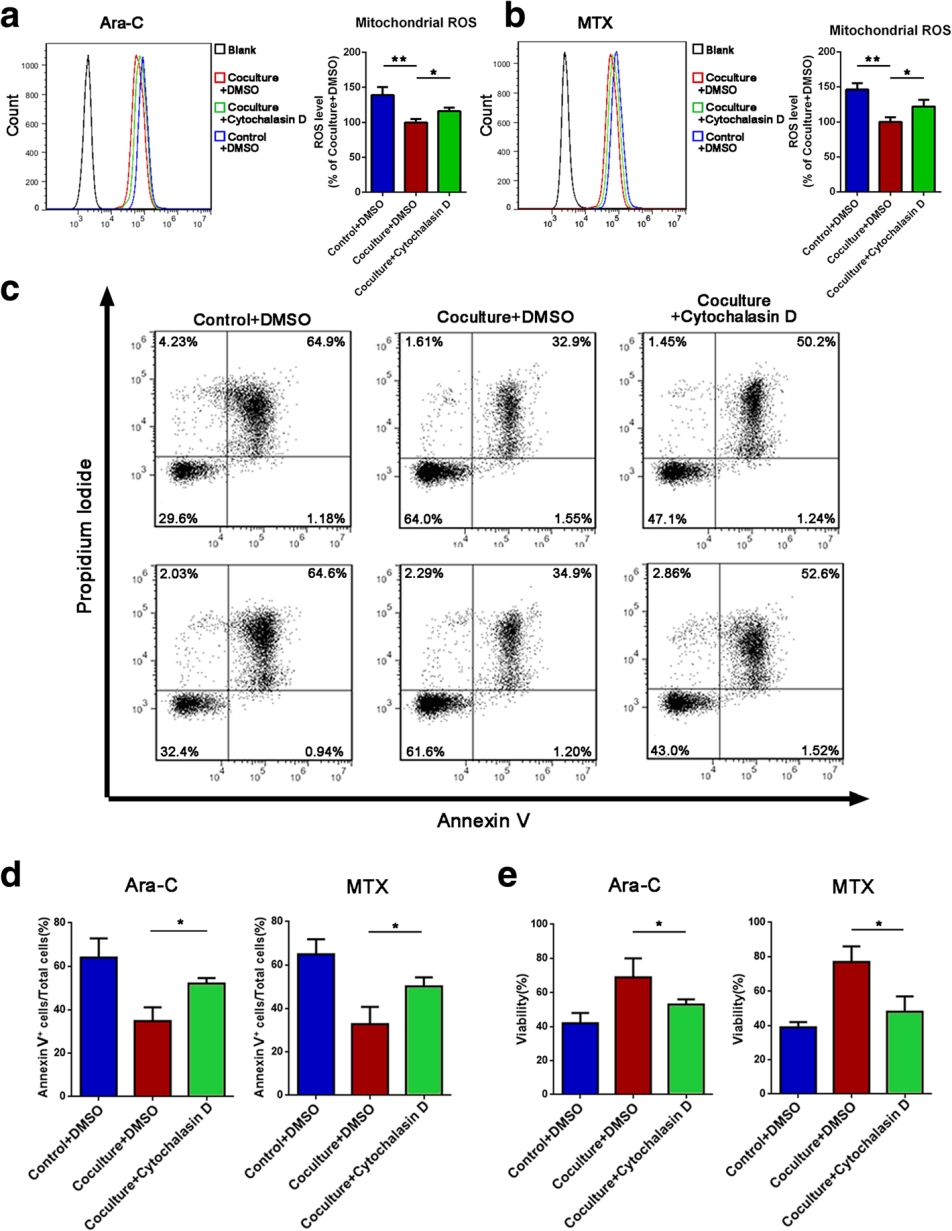
Inhibition of mitochondria transfer decreases the effects of MSC-induced chemoresistance. **a**, **b** Cytochalasin D (1 μM) was added to the coculture system with ara-C or MTX for 48 h. The levels of mitochondrial ROS in Jurkat cells were examined by MitoSOX staining. **c** The apoptosis rate was determined using annexin V/PI staining and FACS. **d** The percentages of annexin V-positive cells were calculated. **e** A CCK-8 assay was used to assess Jurkat cell viability. The data above are presented as the mean ± S.E.M. of three independent experiments (*P < 0.05; **P < 0.01; *t* test)

We next examined whether mitochondria transfer could lead to decreased ROS levels and drug resistance in Jurkat cells. First, we analyzed the mitochondrial ROS levels in Jurkat cells in the coculture system, which showed reduced ROS comparing with non-coculture control group, and treatment with cytochalasin D increased the mitochondrial ROS levels in Jurkat cells upon exposure to ara-C or MTX (Fig. 3a, b). And the mitochondrial DNA damage caused by chemotherapeutics also decreased after coculture (Additional file 1: Figure S2). We further performed annexin V/PI flow cytometry analysis and a cell viability assay and found that Jurkat cells treated with cytochalasin D had an increased apoptosis rate (Fig. 3c, d) and decreased cell viability (Fig. 3e), indicating that blocking mitochondria transfer decreased the capacity of MSCs to protect Jurkat cells from drug cytotoxicity. Although there are slight differences in numerical values between CCK-8 and annexin V/PI results, which may be due to different processing methods, the tendencies consist with each other and both support the conclusions. Taken together, these results demonstrate that mitochondria transfer contributes to the MSC-induced chemoresistance of Jurkat cells.

### ICAM-1-mediated Jurkat cell/MSC adhesion contributes to MSC-induced chemoresistance

**Fig. 4:**
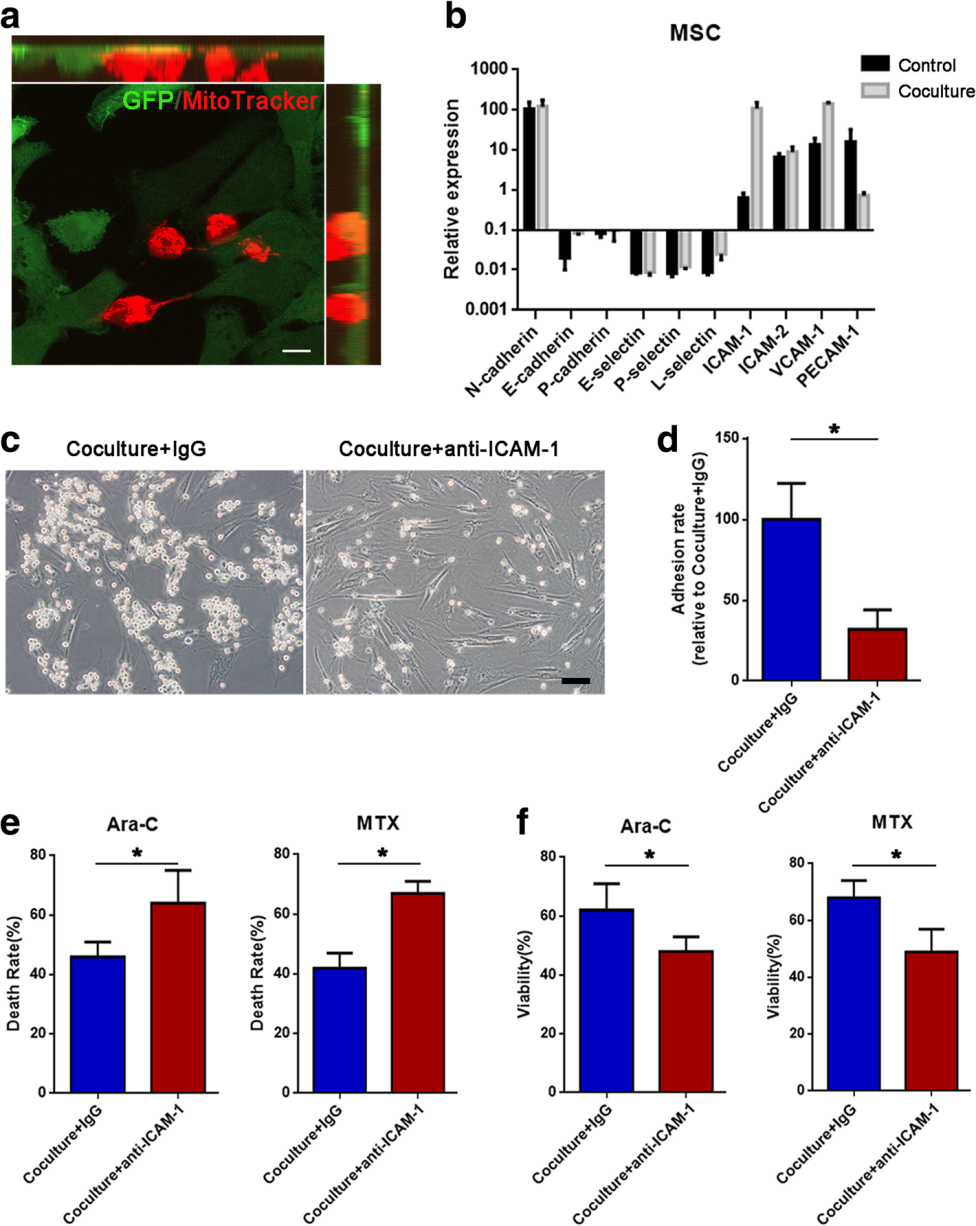
Increased expression of ICAM-1 in MSCs facilitates cell adhesion and protects Jurkat cells from chemotherapeutic drugs. **a** Confocal top view and side view of Jurkat cells (prestained with MitoTracker Red) and GFP-labeled MSCs in a coculture system. Scale bar, 10 μm. **b** mRNA expression of adhesion molecules in MSCs cocultured with Jurkat cells. **c** Representative photos of Jurkat cells and MSCs in the coculture system after removal of the nonadhesive Jurkat cells. Scale bar, 50 μm. **d** The relative adhesion ratio was calculated as the ratio of the number of Jurkat cells adhered to MSCs in the anti-ICAM-1 treated group to that in the DMSO-treated group. **e** The death rate of Jurkat cells was examined by FACS. **f** A CCK-8 assay was used to assess Jurkat cell viability. The data above are presented as the mean ± S.E.M. of three independent experiments (*P < 0.05; **P < 0.01; *t* test)

Intriguingly, we found that most Jurkat cells adhered to MSCs in the direct coculture system. Furthermore, we performed confocal stacking with *z*-spacing and analyzed side views and found that Jurkat cells adhered to MSCs closely (Fig. 4a). To determine which molecules were involved in the adhesion, we analyzed the major adhesion molecules for T cells including cadherins (N-cadherin, E-cadherin, and P-cadherin), selectins (E-selectin, P-selectin, and L-selectin), and the Ig family (ICAM-1, ICAM-2, VCAM-1, and PECAM-1) [44–46]. As shown in Fig. 4b, the mRNA level of ICAM-1was strikingly induced in MSCs cocultured with Jurkat cells, whereas the other adhesion molecules did not show significant changes. We further tested the role of ICAM-1 in Jurkat cell/MSC adhesion using a blocking antibody against ICAM-1(anti-ICAM-1). The results demonstrated that treatment with 20 mg/ml anti-ICAM-1 significantly decreased the number of adhering Jurkat cells (Fig. 4c, d), indicating that ICAM-1 was crucial for cell adhesion between Jurkat cells and MSCs. We further explored whether Jurkat cell/MSC adhesion contributed to MSC-induced chemoresistance. In the coculture system with ara-C or MTX, we also tested whether MSC-induced chemoresistance could be influenced by anti-ICAM-1. We observed that Jurkat cells exposed to anti-ICAM-1 showed an increased cell death rate (Fig. 4e) and decreased cell viability (Fig. 4f). Taken together, these results indicate that ICAM-1-mediated Jurkat cell/MSC adhesion contributes to MSC-induced chemoresistance.

### Mitochondria transfer from Jurkat cells to MSCs is mediated by Jurkat cell/MSC adhesion

**Fig. 5:**
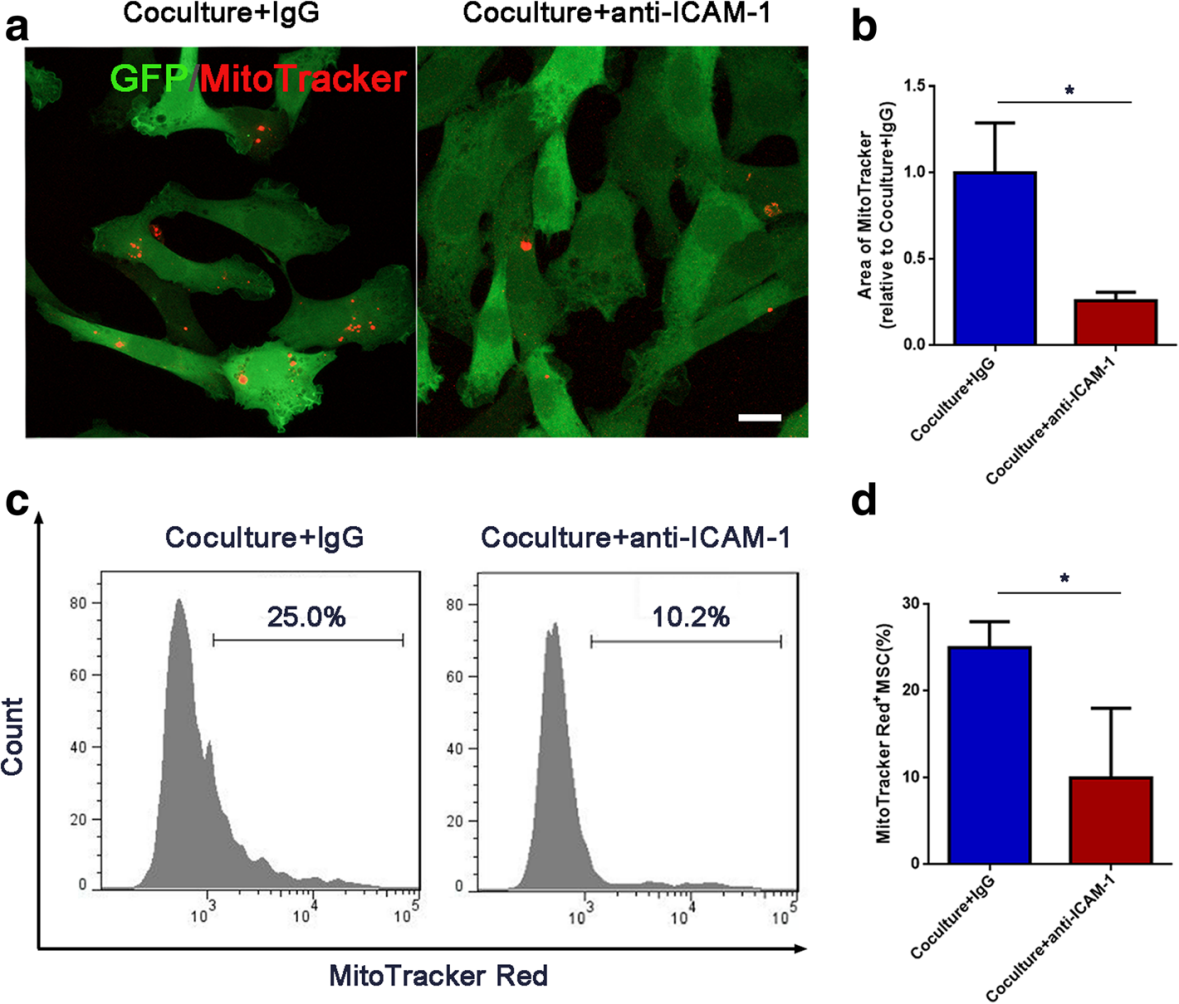
Cell-cell adhesion mediates mitochondria transfer from Jurkat cells to MSCs. Anti-ICAM-1 (20 mg/ml) was added to the coculture system with MTX for 48 h. **a** Confocal images showed that mitochondria transfer was inhibited by anti-ICAM-1. Scale bar, 10 μm. **b** The areas of red foci per field were calculated by ImageJ software. **c** Flow cytometry analysis of MitoTracker Red uptake by MSCs (GFP+ population) cocultured with MitoTracker Red-labeled Jurkat cells for 48 h. **d** The percentage of Red+ MSCs in each group was analyzed and graphed. The data above are presented as the mean ± S.E.M. of three independent experiments (*P < 0.05; **P < 0.01; *t* test)

Based on our finding that ICAM-1-mediated Jurkat cell/MSC adhesion contributed to MSC-induced chemoresistance in Jurkat cells, we explored whether this adhesion contributed to chemoresistance through mitochondria transfer. Jurkat cell/MSC adhesion was blocked by anti-ICAM-1, and then, mitochondria transfer was analyzed with confocal microscopy and flow cytometry. Before coculture, Jurkat cells were stained with MitoTracker Red to label mitochondria. Mitochondria transfer from Jurkat cells to MSCs was obviously inhibited by anti-ICAM-1 treatment (Fig. 5a, b), which was further confirmed by flow cytometry analysis (Fig. 5c, d). On the other hand, we measured the ROS levels in MSCs after coculture with Jurkat cells. With the transfer of mitochondria from Jurkat cells to MSCs, the ROS levels in MSCs increased, so that the metabolic activity of MSCs also increased (Additional file 1: Figure S3). Taken together, these results indicate that cell adhesion between Jurkat cells and MSCs facilitates the process of mitochondria transfer in the presence of chemotherapeutic drugs.

### MSC-induced chemoresistance of primary T-ALL cells is ameliorated by inhibiting TNT formation

**Fig. 6:**
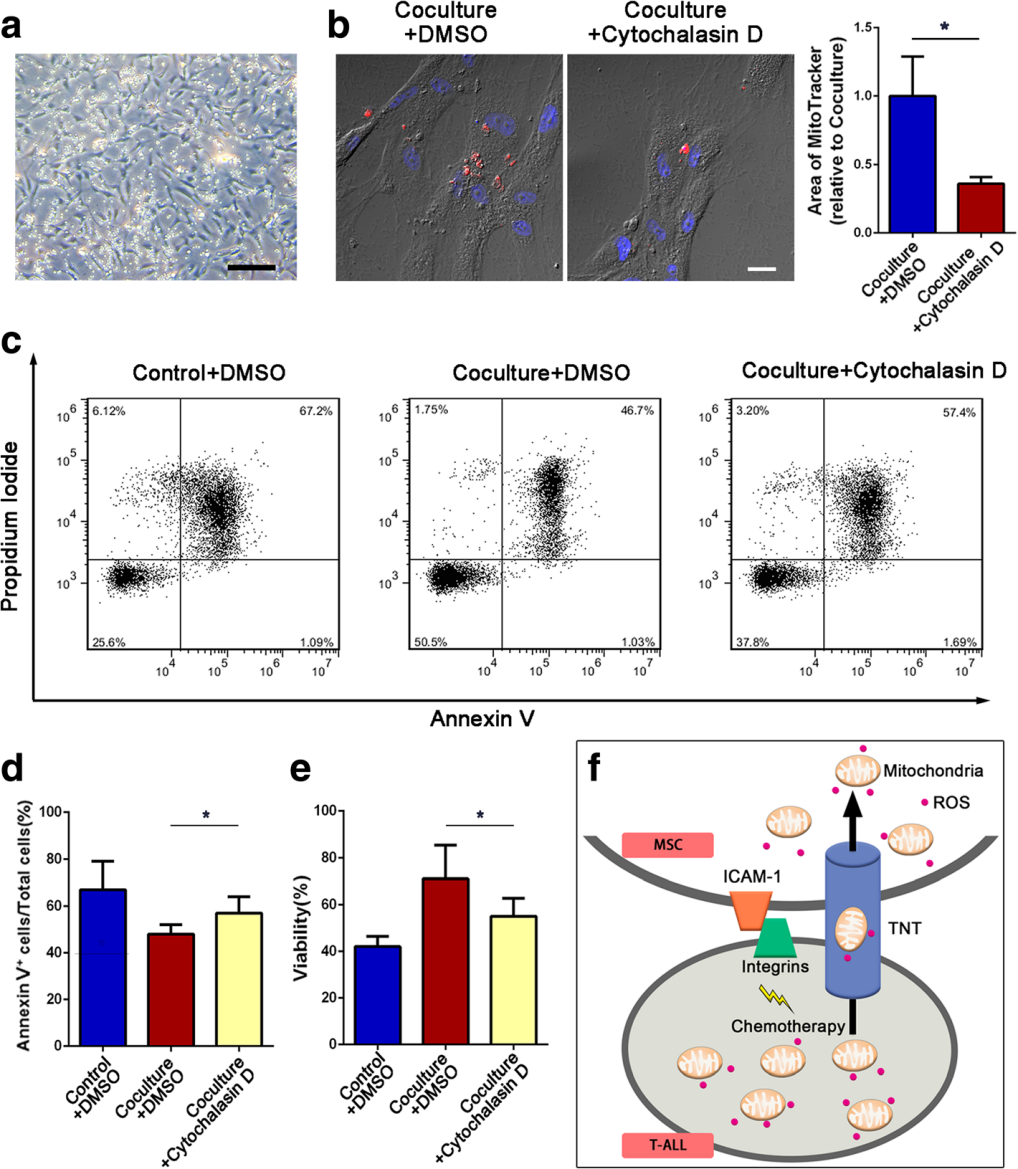
Inhibition of TNT formation ameliorates MSC-induced chemoresistance on primary T-ALL cells. Cytochalasin D (1 μM) was added to the coculture system with ara-C or MTX for 48 h. **a** Representative photos of human primary T-ALL cells and MSCs in the coculture system after the removal of nonadhesive human primary T-ALL cells. Scale bar, 100 μm. **b** Representative confocal microscopy images show that human primary T-ALL cell-derived mitochondria (Red+) were internalized in MSCs. Scale bar, 20 μm. The areas of red foci per field were calculated by ImageJ software. **c** The apoptosis rate was determined using annexin V/PI staining and FACS. **d** The percentages of annexin V-positive cells were calculated. **e** A CCK-8 assay was used to assess human primary T-ALL cell viability. **f** Graphic abstract: T-ALL cell/MSC adhesion-mediated mitochondria transfer contributes to MSC-induced chemoresistance. The data above are presented as the mean ± S.E.M. of three independent experiments (*P < 0.05; **P < 0.01; *t* test)

Finally, we asked whether our findings in Jurkat cells could be recapitulated in human primary T-ALL cells. We labeled human primary T-ALL cells with MitoTracker Red before coculture with MSCs. After 2 days of coculture with ara-C or MTX, some primary human T-ALL cells had adhered to MSCs (Fig. 6a); confocal imaging showed that mitochondrial Red fluorescence was internalized in MSCs. The areas of red foci in MSCs significantly decreased when cytochalasin D was added to the coculture system (Fig. 6b), indicating that mitochondria transfer was impaired when TNT formation was blocked. Similarly, the areas of red foci in MSCs also decreased when anti-ICAM-1 was added (Additional file 1: Figure S4). To further examine the exact effect of cytochalasin D on the survival of human primary T-ALL cells, we performed annexin V/PI flow cytometry analysis and a cell viability assay, and the results were consistent with our cell line experiments. Human primary T-ALL cells cocultured with MSCs and treated with cytochalasin D had a higher apoptosis rate (Fig. 6c, d) and lower cell viability (Fig. 6e) than those cocultured with MSCs only. These results indicate that blocking mitochondria transfer by inhibiting TNT formation decreases the capacity of MSCs to protect T-ALL cells from drug cytotoxicity.

## Discussion

As one of the most aggressive hematologic malignancies, T-ALL is usually treated with multiple chemotherapeutic drugs clinically, but observations of primary drug resistance during treatment are quite frequent. Although it is widely accepted that MSCs are involved in the pro-survival effects, the exact role of MSCs under the chemotherapy remains unclear. Here, we demonstrated that upon the induction of oxidative stress by chemotherapeutic drugs, T-ALL cells were able to transfer mitochondria to MSCs. This process was mediated by TNTs and ICAM-1 contributing to the cell adhesion-mediated drug resistance. A graphical abstract is shown to describe this mechanism briefly (Fig. 6f).

MSCs can trigger the drug resistance of tumor cells via two main strategies, soluble factor-mediated drug resistance and cell adhesion-mediated drug resistance [47]. For the former, drug resistance can be triggered by MSCs secreting cytokines, chemokines, growth factors [27], and exosomes [48], and for the latter, MSCs can induce drug resistance by adhering to cancer cells, including melanoma cells [40] and leukemia cells [49]. In this study, we found that mitochondria transfer between MSCs and T-ALL cells is also a mechanism that induces chemoresistance in tumor cells. According to the literature, intercellular mitochondria transfer can be mediated by TNTs, microvesicles, or gap junctions. We confirmed that TNTs played a major role in the mitochondria transfer between MSCs and T-ALL cells, and inhibition of TNTs led to decreased MSC-induced chemoresistance. This finding may thus provide a novel strategy for T-ALL treatment.

Many studies have demonstrated that the intercellular transferred mitochondria were still functional and can affect cellular fate. For example, mitochondria transferred from MSCs to tumor cells could increase the oxidative phosphorylation and ATP production [47]. Some cancer cells could also induce stromal cells to produce oncometabolites to fuel their metabolism through mitochondria transfer [50]. Meanwhile, it is reported that mitochondrial loss in MSCs could decrease ATP concentrations in these cells, thereby decreasing their secretory capacity and interfering the cytokine secretion which played an important role in maintaining the microenvironment [28]. On the other hand, MSCs might eliminate the transferred mitochondria to stabilize the intracellular homeostasis. Phinney et al. firstly figured out that MSCs eliminated their damaged mitochondria by exporting them to neighboring macrophages for recycling [51], so as to decrease the oxidative pressure in the microenvironment, associated with better survival and increased regrowth potential. In our study, mitochondria transfer helps reduce the ROS level in Jurkat cells, so as to induce chemoresistance. Taken together, investigating the fate of transferred mitochondria helps to understand the crosstalk in leukemic microenvironment and offers probable therapy strategies.

In contrast to the myriad reports demonstrating that MSCs could transfer mitochondria to various kinds of cells, including cortical neurons [52], cardiomyocytes [53], renal tubular cells [30], lung epithelium cells [28], lung adenocarcinoma cells [32], osteosarcoma cells [54], macrophage [51], and acute myeloid leukemia cells [55], mitochondria transfer from other cells to MSCs has rarely been demonstrated. In our study, we found that T-ALL cells could transfer mitochondria to adhering MSCs when treated with ara-C or MTX. This newly identified process complements the existing knowledge of mitochondria transfer and provides a novel perspective regarding mitochondria transfer in intercellular relationships. On the other hand, in cancers such as acute myeloid leukemia (AML), MSCs are impaired in their growth properties and osteogenic differentiation potential [56]. Interestingly, here, we demonstrated that MSCs could receive mitochondria from T-ALL cells, and it is likely that this transfer would lead to MSC damage in T-ALL patients. Since MSCs play an important role in tissue repair [57, 58], they are worthy of further investigation. Moreover, it is reported that AML cells can import mitochondria from MSCs in order to better withstand chemotherapy [55, 59]. Thus, we verified our conclusion by comparing ALL cells with AML cells and found they have different adhesive capacity and mitochondria transfer direction (Additional file 1: Figure S5). The difference in transfer direction may also be due to their different metabolic state. T-ALL cells prefer glycolysis after coculture, while AML cells have more oxidative phosphorylation [60]. ALL cells export mitochondria to reduce intracellular ROS, while AML cells import mitochondria for the demand of oxidative phosphorylation.

Disrupted oxidative stress metabolism is a common feature of cancer cells [61, 62], and this phenomenon has also been observed in T-ALL cells [63]. As a result, ROS levels are higher in T-ALL cells than in non-leukemic cells. Since excess ROS can lead to leukemia cell deaths, the induction of intracellular oxidative stress has been shown to be an important anti-cancer mechanism of leukemia chemotherapy [64]. Thus, the promotion of mitochondrial ROS production can be observed in T-ALL cells treated with paclitaxel, anthracyclines, ara-C, and MTX, among others*.* In our previous study, we found that MSC-mediated chemoresistance of T-ALL cells was dependent on decreased mitochondrial ROS in T-ALL cells, and the ERK/Drp1 signaling pathway was involved in the downregulation of ROS levels. However, mitochondrial ROS in T-ALL cells decreased to a larger extent in a coculture system than in a Transwell system, indicating that an unknown mechanism mediated the MSC-induced chemoresistance. Here, we found that cell adhesion-mediated mitochondria transfer from T-ALL cells to MSCs can reduce oxidative stress by decreasing mitochondrial ROS. This finding solved the question raised by our previous study. Additionally, Ishikawa et al. observed the direct transfer of intercellular ROS mediated by connexin-43 from hematopoietic stem cells to bone marrow stromal cells [65]. This finding suggested that the direct transfer of mitochondrial ROS via TNTs might also be another mechanism for decreasing ROS in T-ALL cells. Unfortunately, due to the limitations of the existing experimental techniques, the two mechanisms have yet to be discriminated.

Additionally, we also found that treatment with anti-ICAM-1 significantly blocked mitochondria transfer, indicating that mitochondria transfer was mediated by T-ALL cell/MSC adhesion. Combined with our finding that blocking mitochondria transfer with cytochalasin D abolished the capacity of MSCs to protect T-ALL cells, we concluded that T-ALL cell/MSC adhesion-mediated mitochondria transfer contributed to MSC-induced chemoresistance. Thus, inhibition of T-ALL cell/MSC adhesion-mediated mitochondria transfer may be a novel strategy for T-ALL treatment.

## Conclusions

Our results elucidate the role of MSCs in the chemoresistance of T-ALL cells. T-ALL cells manage chemotherapy-induced intracellular oxidative stress by targeting mitochondria transfer to MSCs which is mediated by TNTs. Adhesion between MSCs and T-ALL cells is frequently observed in coculture systems. ICAM-1 is the major adhesion molecule. Our results demonstrate that this mechanism should be considered in future clinical investigations. Targeting mitochondria transfer may be a potential strategy for the chemoresistance of T-ALL. Hopefully, this study will serve as a precedent for applying similar therapeutic strategies in the chemoresistance of other leukemias.

## Additional file

Additional file 1: Table S1. Primer used to amplify the human transcripts during real-time quantitative PCR. **Figure S1.** Verification of the feasibility of mitochondria dye method. **Figure S2.** Mitochondrial DNA damage caused by drug-induced ROS in Jurkat cells. **Figure S3.** ROS levels in MSCs increase after coculture with Jurkat cells. **Figure S4.** Anti-ICAM-1 decreases mitochondria transfer between human primary T-ALL cells and MSCs. **Figure S5.** MSCs export mitochondria to AML cells (HL-60 cells) but not ALL cells (Jurkat cells) under chemotherapy. (DOCX 3488 kb)

## Abbreviations

ALL: Acute lymphoblastic leukemia
AML: Acute myeloid leukemia
ANOVA: Analysis of variance
CCK-8: Cell counting kit-8
cDNA: Complementary deoxyribonucleic acid
CLL: Chronic lymphocytic leukemia
FACS: Fluorescence-activated cell sorting
FBS: Fetal bovine serum
GFP: Green fluorescent protein
ICAM-1: Intercellular adhesion molecule -1
MSCs: Mesenchymal stem cells
PECAM-1: Platelet endothelial cell adhesion molecule-1
PI: Propidium iodide
qRT-PCR: Quantitative real-time polymerase chain reaction
ROS: Reactive oxygen species
T-ALL: T cell acute lymphoblastic leukemia
TNTs: Tunneling nanotubes
VCAM-1: Vascular cell adhesion molecule-1
