# Supplementary material for: Cell adhesion-mediated mitochondria transfer contributes to mesenchymal stem cell-induced chemoresistance on T cell acute lymphoblastic leukemia cells
Source: J Hematol Oncol. 2018 Jan 22;11:11. doi: 10.1186/s13045-018-0554-z (PMC5778754; doi:10.1186/s13045-018-0554-z)
Supplement: Additional file 1: Table S1. — Primer used to amplify the human transcripts during real-time quantitative PCR. Figure S1. Verification of the feasibility of mitochondria dye method. Figure S2. Mitochondrial DNA damage caused by drug-induced ROS in Jurkat cells. Figure S3. ROS levels in MSCs increase after coculture with Jurkat cells. Figure S4. Anti-ICAM-1 decreases mitochondria transfer between human primary T-ALL cells and MSCs. Figure S5. MSCs export mitochondria to AML cells (HL-60 cells) but not ALL cells (Jurkat cells) under chemotherapy. (DOCX 3488 kb) [file 13045_2018_554_MOESM1_ESM.docx]

**Additional file 1**

**Cell adhesion-mediated mitochondria transfer contributes to mesenchymal stem cells-induced chemoresistance on T-cell acute lymphoblastic leukemia cells**

Jiancheng Wang, Xin Liu, Yuan Qiu, Yue Shi, Jianye Cai, Boyan Wang, Xiaoyue Wei, Qiong Ke, Xin Sui, Yi Wang, Yinong Huang, Hongyu Li, Tao Wang, Ren Lin, Qifa Liu, Andy Peng Xiang

*Correspondence: Dr Andy Peng Xiang, Center for stem cell biology and tissue engineering, Sun Yat-Sen University, 74# Zhongshan 2nd Road, Guangzhou, Guangdong, China. Phone: 86-20-87335822; Fax: 86-20-87335858; E-mail: xiangp@mail.sysu.edu.cn

| **Table S1.** **Primer used to amplify the human transcripts during real-time quantitative PCR.** | | | |
| --- | --- | --- | --- |
| **Gene** | **Sequence (5′ to 3′)** | **Application** | |
| **N-cadherin**  **(Human)** | **Upper: AGC CAA CCT TAA CTG AGG AGT**  **Lower: GGC AAG TTG ATT GGA GGG ATG** | | **qRT-PCR** |
| **E-cadherin**  **(Human)** | **Upper: CGA GAG CTA CAC GTT CAC GG**  **Lower: GGG TGT CGA GGG AAA AAT AGG** | | **qRT-PCR** |
| **P-cadherin**  **(Human)** | **Upper: ATC ATC GTG ACC GAC CAG AAT**  **Lower: GAC TCC CTC TAA GAC ACT CCC** | | **qRT-PCR** |
| **E-selectin**  **(Human)** | **Upper: CAG CAA AGG TAC ACA CAC CTG**  **Lower: CAG ACC CAC ACA TTG TTG ACT T** | | **qRT-PCR** |
| **P-selectin**  **(Human)** | **Upper: ACT GCC AGA ATC GCT ACA CAG**  **Lower: CAC CCA TGT CCA TGT CTT ATT GT** | | **qRT-PCR** |
| **L-selectin**  **(Human)** | **Upper: ACC CAG AGG GAC TTA TGG AAC**  **Lower: GCA GAA TCT TCT AGC CCT TTG C** | | **qRT-PCR** |
| **ICAM-1**  **(Human)** | **Upper: ATG CCC AGA CAT CTG TGT CC**  **Lower: GGG GTC TCT ATG CCC AAC AA** | | **qRT-PCR** |
| **ICAM-2**  **(Human)** | **Upper: CGG ATG AGA AGG TAT TCG AGG T**  **Lower: CAC CCA CTT CAG GCT GGT TAC** | | **qRT-PCR** |
| **VCAM-1**  **(Human)** | **Upper: GGG AAG ATG GTC GTG ATC CTT**  **Lower: TCT GGG GTG GTC TCG ATT TTA** | | **qRT-PCR** |
| **PECAM-1**  **(Human)** | **Upper: AAC AGT GTT GAC ATG AAG AGC C**  **Lower: TGT AAA ACA GCA CGT CAT CCT T** | | **qRT-PCR** |
| **β-actin**  **(Human)** | **Upper: ACT TAG TTG CGT TAC ACC**  **Lower: AAT CCT GAG TCA AGC CAA** | | **qRT-PCR** |

**Supplementary Material and Methods**

**Measurement of oxidative mtDNA damage**

The level of mtDNA damage was measured by assessing the relative levels of mtDNA-ND1 to 8.9-kb mitochondria fragment using a qRT-PCR analysis of DNA extracted from Jurkat cells.

The following amplification primers were used (5’ to 3’): mtDNA-ND1 (sense, CCCTAAAACCCGCCACATCT; antisense, GAGCGATGGTGAGAGCTAAGGT) and

8.9-kb mitochondria fragment (sense, TCTAAGCCTCCTTATTCGAGCCGA; antisense, TTTCATCATGCGGAGATGTTGGATGG).

**Measurement of intracellular ROS accumulation**

CellROX Deep Red Reagent (Invitrogen) is a ﬂuorogenic probe designed to reliably measure ROS in living cells. MSCs were incubated with CellROX™ Deep Red Reagent (5 µM) at 37°C for 30 min with protection from light. Subsequently, medium was removed, and the cells were washed three times with PBS. The resulting ﬂuorescence was measured using a Laser Scanning Confocal Microscope (LSM800; Zeiss). Mean fluorescent intensity per cell was quantified using NIH image J program.

**Fluorescence assay of mitochondria transfer**

MitoTracker Red (Molecular Probes) was used to label mitochondria. MSCs were incubated with 200 nM MitoTracker Red in culture media for 10 min at 37 °C. Excess of the dye was washed out with PBS. Stained cells were then seeded for coculture with Jurkat cells or HL-60 cells 4 days later. The resulting ﬂuorescence was measured using a Laser Scanning Confocal Microscope (LSM800; Zeiss).


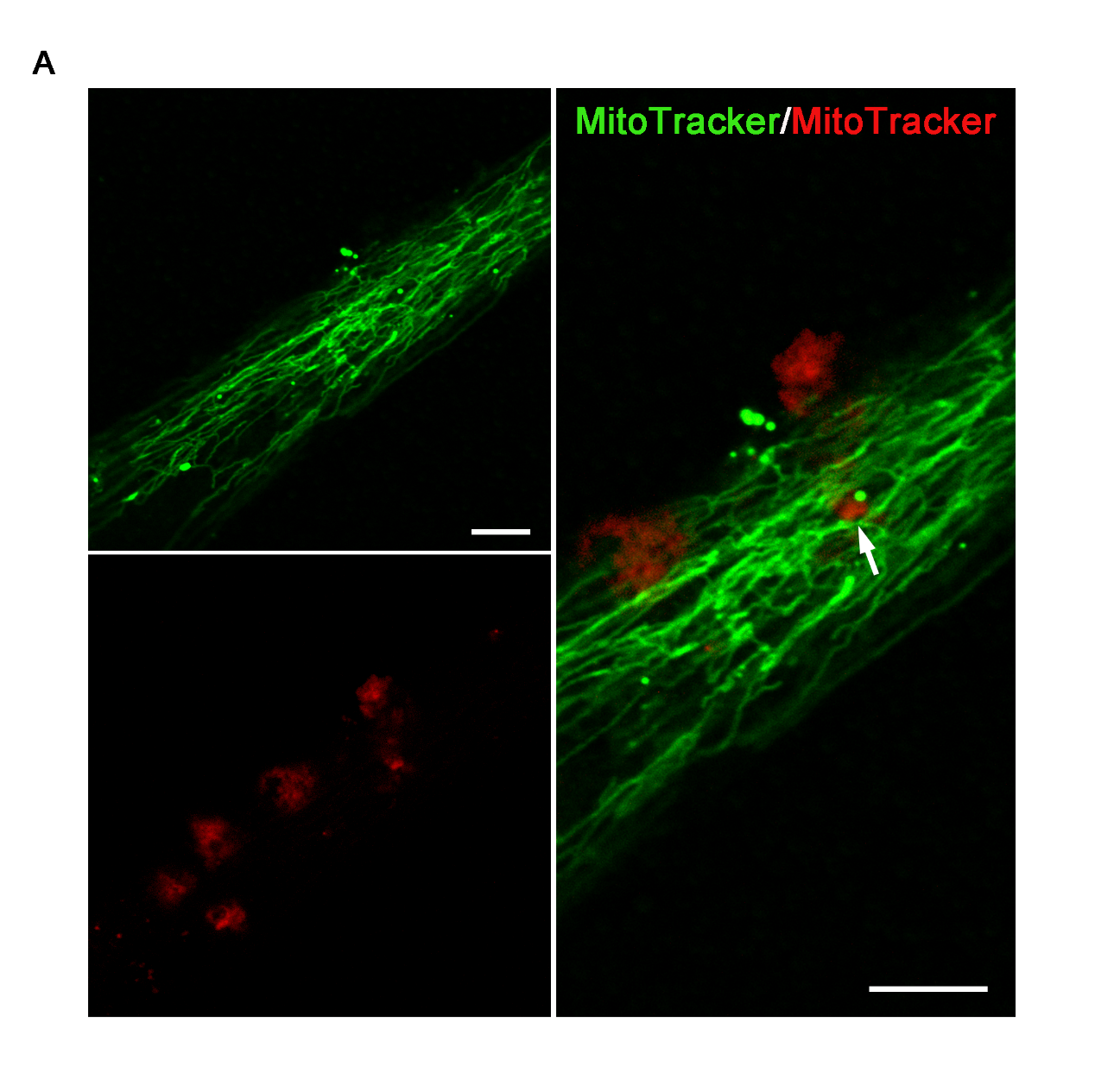


Figure S1. Verification of the feasibility of mitochondria dye method. Description of data: (A) Mitochondria (arrow) were transferred to MSCs from Jurkat cells after coculture, but there was no merged color in the mitochondria of MSCs. Green: mitochondria in MSCs; Red: mitochondria in Jurkat cells and from Jurkat cells. Scale bar, 10 μm. The data above are presented by three independent experiments.





Figure S2: Mitochondrial DNA damage caused by drug-induced ROS in Jurkat cells. Description of data: (A) Relative mtDNA damage of Jurkat cells in coculture system decreased compared to control groups. The data above are presented as the mean±S.E.M. of three independent experiments. (*P<0.05; **P<0.01; t-test)


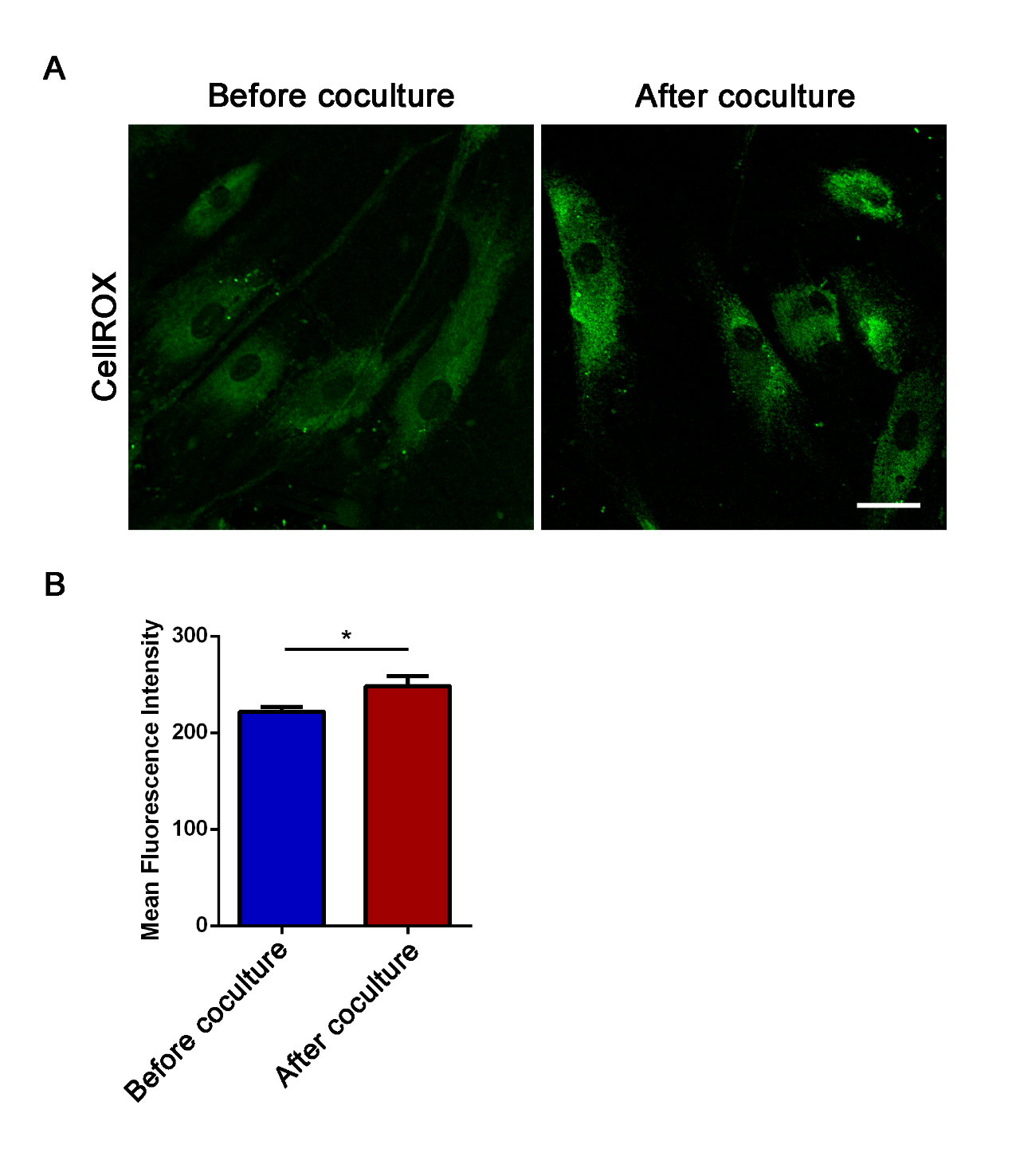


Figure S3. ROS levels in MSCs increase after coculture with Jurkat cells. Description of data: (A) Confocal images showed MSCs have more ROS after coculture with Jurkat cells. Scale bar, 50 μm. (B) Mean fluorescent intensity per cell was quantified using NIH image J program. (C) A CCK-8 assay was used to assess MSC viability. The data above are presented as the mean±S.E.M. of three independent experiments. (*P<0.05; **P<0.01; t-test)


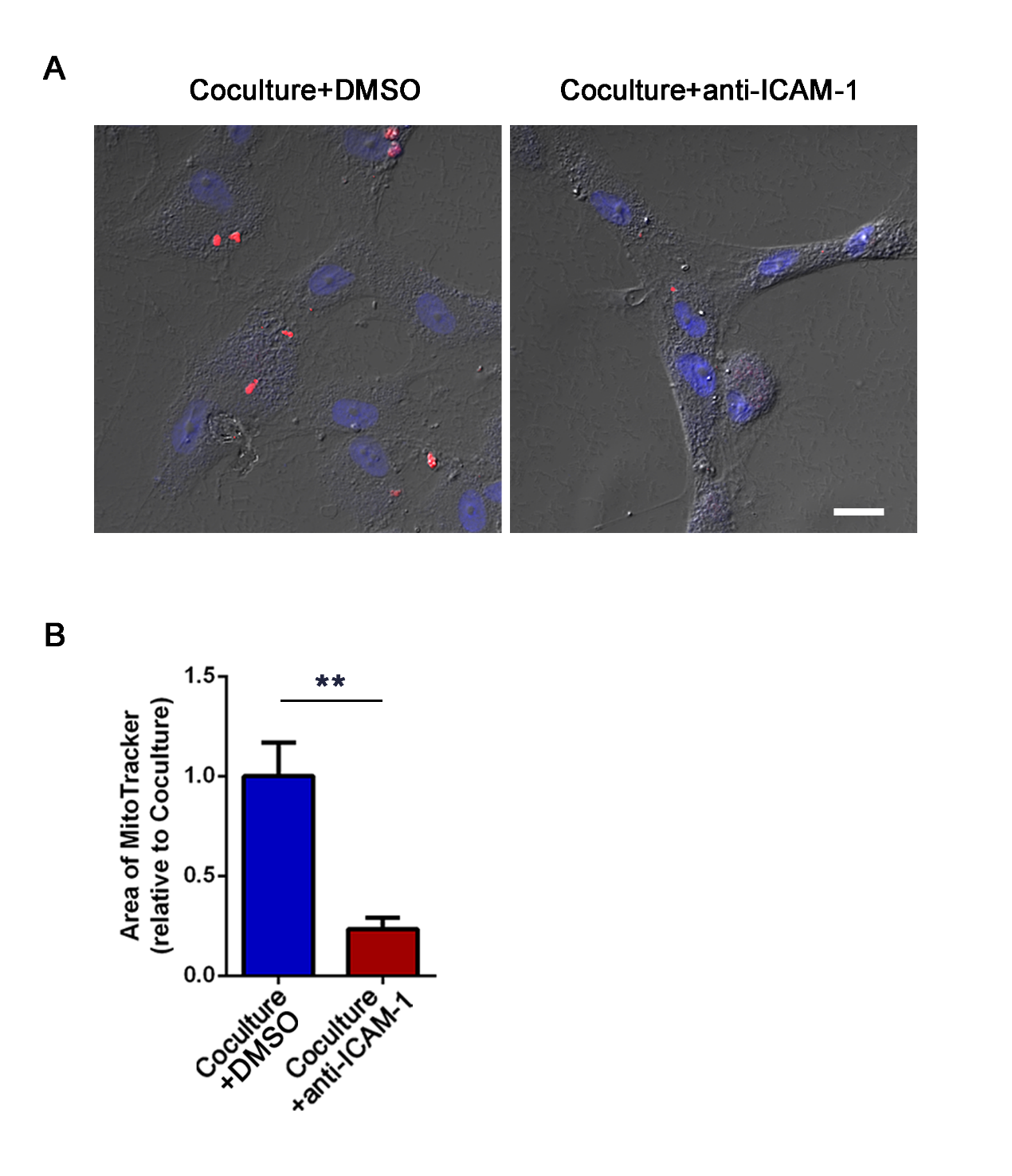


Figure S4. Anti-ICAM-1 decreases mitochondria transfer between human primary T-ALL cells and MSCs. Description of data: (A) Representative confocal microscopy images show that human primary T-ALL cell-derived mitochondria (Red+) were internalized in MSCs. Scale bar, 20μm. (B) The areas of red foci per field were calculated by ImageJ software. The data above are presented as the mean±S.E.M. of three independent experiments. (*P<0.05; **P<0.01; t-test)


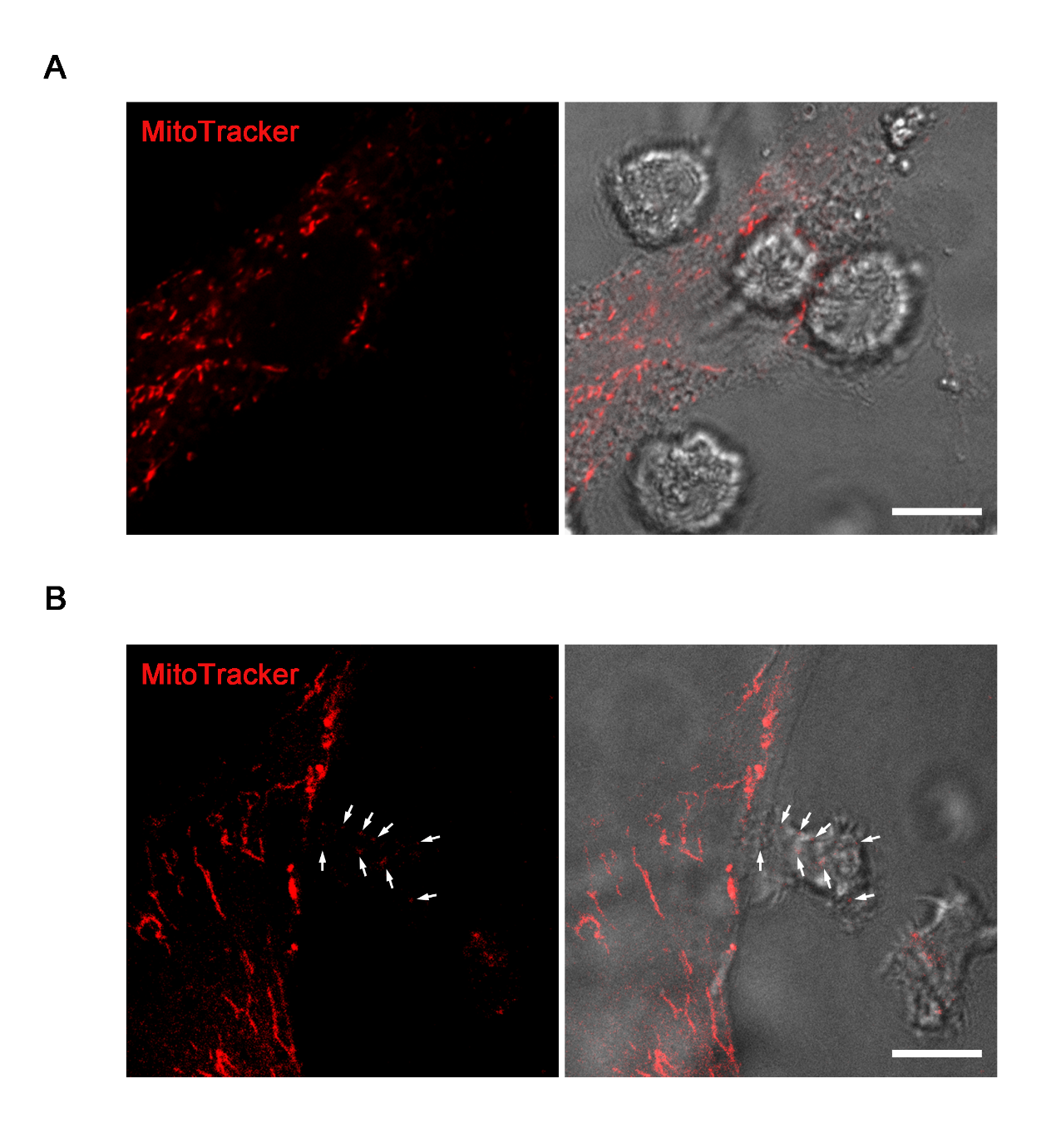


Figure S5. MSCs export mitochondria to AML cells (HL-60 cells) but not ALL cells (Jurkat cells) under chemotherapy. Description of data: (A) Confocal images showed almost no mitochondria transferred from MSCs to ALL cells. Scale bar, 10 μm. (B) Confocal images showed AML cells import mitochondria (arrow) from MSCs. Scale bar, 10 μm. The data above are presented by three independent experiments.
